# Personalized Shared Control for Automated Vehicles Considering Driving Capability and Styles

**DOI:** 10.3390/s24247904

**Published:** 2024-12-11

**Authors:** Bohua Sun, Yingjie Shan, Guanpu Wu, Shuai Zhao, Fei Xie

**Affiliations:** 1 College of Automotive Engineering, the National Key Laboratory of Automotive Chassis Integration and Bionics, Jilin University, Changchun 130025, China; shanyj23@mails.jlu.edu.cn (Y.S.); xiefei@jlu.edu.cn (F.X.); 2 College of Intelligence and Computing, Tianjin University, Tianjin 300354, China; zhaoshuai@catarc.ac.cn; 3 Automotive Data Center, CATARC, Tianjin 300000, China

**Keywords:** automated vehicle, driving capability, driving style, human–machine hybrid, decision-making, shared control

## Abstract

The shared control system has been a key technology framework and trend, with its advantages in overcoming the performance shortage of safety and comfort in automated vehicles. Understanding human drivers’ driving capabilities and styles is the key to improving system performance, in particular, the acceptance by and adaption of shared control vehicles to human drivers. In this research, personalized shared control considering drivers’ main human factors is proposed. A simulated scenario generation method for human factors was established. Drivers’ driving capabilities were defined and evaluated to improve the rationality of the driving authority allocation. Drivers’ driving styles were analyzed, characterized, and evaluated in a field test for the intention-aware personalized automated subsystem. A personalized shared control framework is proposed based on the driving capabilities and styles, and its evaluation criteria were established, including driving safety, comfort, and workload. The personalized shared control system was evaluated in a human-in-the-loop simulation platform and a field test based on an automated vehicle. The results show that the proposed system could achieve better performances in terms of different driving capabilities, styles, and complex scenarios than those only driven by human drivers or automated systems.

## 1. Introduction

Automated vehicles have become a dominant trend in improving traffic safety and transportation efficiency, given their advantages in reducing drivers’ driving loads and predicting traffic situations [1,2]. However, some obstacles still exist in enhancing the safety and adaptability of fully automated vehicles. Social dilemmas, represented by the tram issue and driver complacency, are technical problems that human society and ethics present to artificial intelligence theory in automated vehicles [3]. The technique limitations of automated vehicles, such as poor perception accuracy and decision-making ability, restrict the transition process as well [4,5]. Therefore, the use of highly automated vehicles with a human-in-the-loop, which is called shared control, is likely to last for a long time and has been attracting increasing attention. Shared control can be defined as a safe, efficient, friendly, and stable driving mode formed by overcoming the decision-making conflict between the driver with social attributes and the automated system with logical attributes. The human–machine cooperation mechanism constitutes the theory foundation of the shared control system, and the human factors are required to be evaluated in detail.

As the key technique to overcoming the human–machine conflict in shared control, the driving authority allocation strategy (DAAS) needs to be analyzed in depth. The DAAS can be defined as the weight distribution method between the driver and the automated system, with the aim of achieving a safe, efficient, and stable system configuration [6]. The DAAS consists of a switched type and a shared type, and the latter is subdivided into direct and indirect shared controls [7]. With the development of by-wire and intelligent sensing techniques, the basic functionality of the DAAS has been improved, and indirect shared control has developed rapidly by virtue of its framework advantage, signal identification, velocity optimization, and H∞ control theories [8,9]. In recent years, in order to improve system safety, adaptability, and driver acceptability, the DAAS has gradually focused on the cognitive mechanism of human factor attributes, the cognitive capability of complex scenarios, and switching smoothness [10,11]. Drivers’ driving skills are modeled and evaluated based on the optimal driver preview model, overtaking model, and accident assessment model and the drivers’ learning processes and levels are revealed, mainly based on typical physical models [12,13]. Drivers’ driving statuses focus on drivers’ fatigue, sleepiness, distraction, and mood and are identified accurately based on biological signals, vehicle states, and driving actions, such as eye and head movements. Image detection and machine learning theories are used to analyze drivers’ take-over and reaction abilities, and they reveal the inducement and externalization of the driving status [14,15]. Drivers’ driving styles are classified and identified based on physical modeling or a data-driven approach, and the technique details are discussed in the next section. It can be seen that key human factors are analyzed and evaluated in a decoupled way, and few studies were conducted on the comprehensive representation of driving skills, statuses, and styles. The rationality of the DAAS still needs to be improved due to the lack of comprehensive human factors reflecting drivers’ time-varying abilities for vehicle control.

As the main component of a human-like automated driving pattern, a personalized driving pattern is produced by a driver’s driving style [16]. Understanding the drivers’ driving styles that make the shared control more human-like is the key to improving system performance, in particular, driver adaption and acceptance [17]. Four human-like degrees, namely, none, low, medium, and high levels, were researched, and the medium degree received more attention based on its advantage of high computational efficiency and distinct personalized results [18]). The classification and identification methods were mainly developed to obtain up to six typical driving styles [19,20]. The classification mode of driving styles is the primary task used to reflect the intrinsic mechanism of the human-like driving pattern and is mainly affected by scenarios, modal datasets, and characteristic parameter sets [21]. No more than five types of driving styles produce optimal computational efficiency but are mainly used for fuel economy instead of drivers’ acceptability [22]. Subjective and objective classification methods were researched individually by online or offline methods, and the average accuracy was about 85%, which was based on data-driven algorithms, and 80% was based on a questionnaire [23]. The identification process of driving styles takes the moment-based array or time-series-based high-dimensional data as the model inputs, and the identification method is mainly based on machine learning and system modeling [19,24]. Data-driven methods have advantages in processing the human factor data with high-order nonlinearity and uncertainty, and the framework of the system model has low-order nonlinearity and certain physical interpretations. Considering the model’s complexity, related methods are mainly conducted using offline methods to obtain analysis results [25]. Furthermore, few studies focused on scenario generation methods specific to human factors, which are the main factors restricting the evaluation accuracy of human factors as well. Therefore, a combination of classification and identification processes for driving styles needs to be developed in depth, and its evaluation method combining online and offline methods and simulated scenarios for the personalized system needs to be established to improve evaluation accuracy.

The decision-making process is required to generate efficient and stable driving tasks in complex scenarios and becomes the core-level component in shared control [26]. The DAAS and driving styles constitute internal constraints and determine the system features together with the external ones, such as scenarios or vehicle states [27]. Thus, the system features of the decision-making process consist of self-learning, high-order nonlinearity, high data dependency, and personality, and the key challenge is how to deal with the uncertainties and improve driver acceptance [28]. Two typical methods, the physical modeling-based and data-driven-based, are researched for decision-making logic. The physical modeling-based methods, such as the car-following model, the Pipes and Forbes model, and the overtaking maneuver model, aim to reveal the decision-making mechanisms of the human–vehicle closed loop system [29]. The model’s accuracy is improved with the gradual refinement of driving skill sub-models, such as the driver preview model, the unified driver model, or the driver control model [30]. The physical modeling-based decision-making models have clear physical meaning and can realize real-time and strong, robust decision-making tasks. However, these models poorly adapt to scenarios with high uncertainty due to the deterministic internal model structure and the neglect of data support. Data-driven-based methods, such as the multi-criteria, Bayesian network, or deep learning decision-making, are trained and operated based on datasets for the online decision-making of automated vehicles [31,32]. Artificial intelligence and research theories, such as game theory, the Markov decision-making process (MDP), or deep learning, are used to examine high-order nonlinearity and personality characteristics occurring in the decision-making process based on large-scale datasets [33]. However, few studies combine intention-aware uncertainty and personalized factors into the decision-making process in shared control, which affects the acceptance and adaption of shared control to a certain extent. As a result, the data-driven-based decision-making paradigm needs to be developed in depth, where the personality of a driver should be merged.

Motivated by the above-mentioned observations, a personalized shared control framework considering a person’s driving capability and style is proposed. The contributions are as follows.

(1) Drivers’ driving capability is defined and evaluated to improve the rationality of the DAAS. Driving styles combining classification and identification processes are analyzed, characterized, and evaluated in both online and offline ways. The simulated scenario generation method for human factors is established.

(2) The personalized framework of shared control for the automated vehicle is proposed, considering drivers’ driving capabilities and style, and its evaluation criteria are established considering driving safety, comfort, and workload. The intention-aware decision-making logic is proposed based on the mixed observable Markov decision process (MOMDP).

## 2. Analysis and Evaluation of Drivers’ Driving Capability and Styles

### 2.1. Simulated Scenario Generation Methods for Human Factors

The system’s simulation and scenario generation method have important significance in improving the rationality and accuracy of evaluation methods and reveal the internal mechanism of human factors, in particular, driving capability and styles. In order to motivate human factors to a maximum extent, the single source-based simulation and the random vehicle–road field (RVRF)-based scenario generation method are proposed for drivers’ driving capability and styles, respectively.

The single source-based simulation consists of signal features, physical mapping, and signal dimension and can be designed as the velocity function of background vehicles based on the signal periodicity, mutability, and spectral characteristics, such as the aperiodic mutation signal and periodic gradient signal as follows:

(1) $$v_{L}=A_{p}\sin\left(\omega t+\phi \right)+\Gamma$$

where *v_L_* is the velocity of the background vehicle, *A_p_* is the velocity amplitude, *ω* is the signal frequency, *φ* is the initial velocity phase, and Γ is the velocity offset.

**Figure 1:**
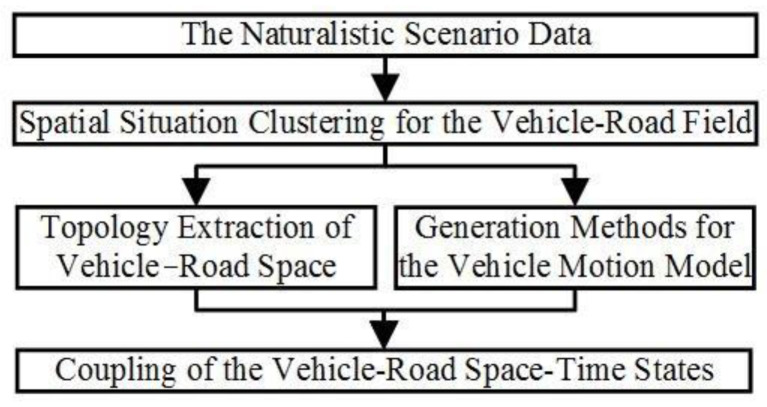
The system framework of RVRF.

Micro-scenarios analyze the physical mechanism and random effect of vehicle–road coupling to reveal a general pattern of microscopic traffic scenarios. Therefore, they are suitable for the evaluation of driving capability, reflecting drivers’ time-varying features. The RVRF can be defined as a strong, random, and steady field formed by the vehicle–road coupling in the simulation environment, and the system framework of RVRF is shown in Figure 1.

The field strength *E_vef_* of RVRF consists of the field strength *E_kef_* of the kinetic energy field composed of moving objects, the *E_ve_*_f_ of the potential field composed of stationary objects, and the *E_vef_* of the intention field composed of drivers’ uncertainty. Three components, generated by the transportation participant P at the spatial point (*x_q_*, *y_q_*), can be expressed as follows:

(2) $$E_{vef}=E_{kef}+E_{pef}+E_{if}$$

(3) $$E_{kef}=\frac{GR_{P}M_{P}}{{\left|r_{Pq}\right|}^{k_{1}}}\frac{r_{Pq}}{\left|r_{Pq}\right|}e^{\left[k_{2}v_{P,lon}\cos(\theta_{P})\right]}$$

(4) $$E_{pef}=\frac{GR_{P}M_{P}}{{\left|r_{Pq}\right|}^{k_{1}}}\frac{r_{Pq}}{\left|r_{Pq}\right|}$$

(5) $$E_{if}=\frac{GR_{P}M_{P}}{{\left|r_{Pq}\right|}^{k_{1}}}\frac{r_{Pq}}{\left|r_{Pq}\right|}e^{\left[k_{2}v_{P,lon}\cos(\theta_{P})\right]}\Phi_{D}$$

where (*G*, *k*_1_, *k*_2_) are constants, *v_P_* is the longitudinal velocity of P, *r_Pq_* is the distance vector at (*x_q_*, *y_q_*), Φ*_D_* is the intention factor, *M_P_* is the equivalent mass, and *R_P_* is road field index.

The RVRF map Ω at the specific moment can be obtained and expressed as follows after slicing the natural scenario data:

(6) $$\Omega =\left\{E_{Svef,\zeta }(x_{\zeta },y_{\zeta }),\zeta =1,\ldots ,n\right\}$$

where *E_Svef,ζ_* is the field strength of the vehicle–road field (*x_ζ_*, *y_ζ_*). Thus, the process of spatial situation clustering is that of extracting the statistical characteristics of the field map and clustering the field map.

Considering the spatial variation feature of the field strength and its neighborhood correlation, the mean neighborhood two-dimensional histogram theory is proposed to extract the statistical characteristics of the field strength. The mean field strength of the N × N neighborhood is centered in *(x_ζ_*, *y_ζ_*).

(7) $$G_{E}(x_{\zeta },y_{\zeta })=\frac{1}{N\times N}\sum_{i=\frac{N-1}{2}}^{\frac{N-1}{2}}\sum_{j=\frac{N-1}{2}}^{\frac{N-1}{2}}E_{Svef,\zeta }(x_{\zeta }+i,y_{\zeta }+j)\;x_{\zeta }\in [1,Q_{x}];y_{\zeta }\in [1,Q_{y}]$$

where *G_E_*(*x_ζ_*, *y_ζ_*) is the average field strength at (*x_ζ_*, *y_ζ_*), and *Q_x_* and *Q_y_* are field map boundaries. The probability of (*E_Svef,ζ_*(*x_ζ_*, *y_ζ_*) = *E_m_*, *G_E_*(*x_ζ_*, *y_ζ_*) = *E_n_*) can be expressed as follows:

(8) $$\begin{array}{l} H_{2D\_mean}(E_{m},E_{n})=P(E_{Svef,\zeta }(x_{\zeta },y_{\zeta }))\\ =\frac{N(E_{Svef,\zeta }(x_{\zeta },y_{\zeta })=E_{m},G_{E}(x_{\zeta },y_{\zeta })=E_{n})}{Q_{x}\times Q_{y}} \end{array}$$

where *H_2D_mean_*(*E_m_*, *E_n_*) is the mean neighborhood histogram. The similarity index *S_I_* is the sum of the minimum values of *H_2D_mean_*, and the spatial situation database *D_S_* is as follows, where *w* is the situation number, and *u* is the field map number.

(9) $$D_{S}=\left\{D_{S,i}=\Omega_{j},i=1,\ldots w,j=1\ldots ,u\right\}$$

The spatial position of the ego vehicle obeys the probability distribution P(*S_t_* = (*S_tx_*, *S_ty_*)), and its set *ξ* is as follows:

(10) $$\xi =\left\{\xi_{i}=(x_{Stx},y_{Stx})\sim P(x_{Stx},y_{Stx}),i=1,\ldots w\right\}$$

where *S_tx_* and *S_ty_* are the numbers of ego vehicle position, and *ξ* is the completely observable set with a high density, fragmentation, and strong randomness in the naturalistic scenario data. Therefore, the growing neutral gas (GNG) algorithm [34] is proposed to extract the vehicle’s continuous topology in *D_S_*, as follows:

(11) GNG={χ,ε}

where *χ* and *ε* are the node set and the boundary set, and the submanifold consists of {*χ*[*i*], *i* = 1, …, *G*} and its eigenvector *ev[i]* = [*χ*[*i*]·*S_tx_, χ*[*i*]·*S_ty_*]^T^ ∈ *R^g^*. *ξ* is taken as the input, and the learning process of *χ* is that of searching the minimum offset as follows, where *D_e_* is the Euclidean distance between *S_t_* and *e_v_*.

(12) $$\chi^{*}=\arg\min\chi \;\sum_{i=1}^{G}\int D_{e}((x_{Stx},y_{Stx}),e_{v}^{\left[i\right]})P(S_{t})dx$$

The velocity of the ego vehicle obeys the specific motion pattern in typical DS, and its spatio-temporal variation features can be generalized by Gaussian process regression (GPR) as follows, where *f*(*X*) is the function of the mean value *η*(*X*) and the covariance *ϑ*(*X*, *X′*).

(13) $$y=f(X)+l,f\sim GP(\eta ,\vartheta ),l\sim N(0,\sigma_{l}^{2})$$

The generation process of the vehicle motion pattern is that of the position searching and the related velocity mapping MP(*x*), and MP:(*S_tx_*, *S_ty_*)^T^ → (*v_tx_*, *v_ty_*)^T^ can be expressed as follows:

(14) $$v_{MP}\sim MP(x)=N({\bar{v}}_{MP},\Sigma_{v_{MP}}),\forall S_{t}^{T}\in \xi$$

The RVRF model H*_ru_* can be abstracted as the probabilistic model consisting of the mutually exclusive region *R_e_*, vehicle–road topology {*X_Re_*, *ε_Re_*}, and the state transition matrix *T_m_* as follows:

(15) Hru={Re,{χRe,εRe},MPRe,Tm}Re={Rei,i=1,…,ϖ,Rei∩Rej=φ,∀i≠j}Tm=P(Rei|Rej,ru)

where the subscript *ru* indicates traffic rules, and the spatial probability distribution P(*S_t_*_+*Δt*_|*R_e_*,*_t_* = Re[*i*]) at the (*t* + Δ*t*) moment can be expressed as follows:

(16) $$\begin{array}{l} P\left(S_{t+\Delta t}\;|R_{e,t}=R_{e}^{\left[i\right]}\right)=\sum_{S_{t}}P(S_{t+\Delta t}\;|S_{t},R_{e}^{\left[i\right]})P\left(S_{t}|R_{e}^{\left[i\right]}\right)\\ =\sum_{S_{t}}\sum_{v_{MP}}\underset{\mathrm{Velocity}\;\mathrm{integral}}{\underset{︸}{P(S_{t+\Delta t}\;|S_{t},v_{MP})}}\underset{GRP}{\underset{︸}{P\left(v_{MP}|S_{t}\right)}}\underset{\mathrm{Observed}\;\mathrm{value}}{\underset{︸}{P\left(S_{t}|R_{e}^{\left[i\right]}\right)}} \end{array}$$

The observation set can be estimated by predicting the vehicle’s spatial distribution, and the maximum likelihood function *u_R,j_* can be expressed as follows, where *M_j_* is the observed value number corresponding to *ψ_t_*_+Δ*t*_^[*j*]^ = 1.

(17) $$u_{R,j}^{\left[i\right]}=M_{j}/M,\forall j\in \left\{1,\ldots ,\varpi \right\}$$

### 2.2. The Mechanism Analysis and Evaluation Method for Driving Capability

**Figure 2:**
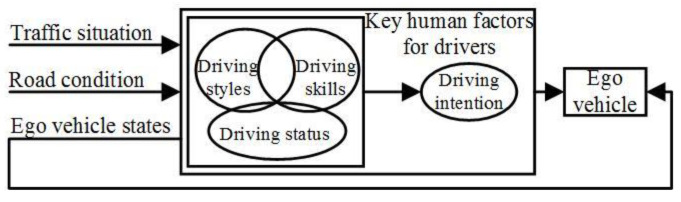
Coupling relationships among human factors.

In order to improve the rationality of the DAAS, coupling relationships among human factors are analyzed and shown in Figure 2. The comprehensive human factor consisting of the driving style, skill, and status is proposed as the driver’s driving capability, which can be defined as the driver’s maneuverability to the vehicle with time-varying nonlinear dynamic features.

**Figure 3:**
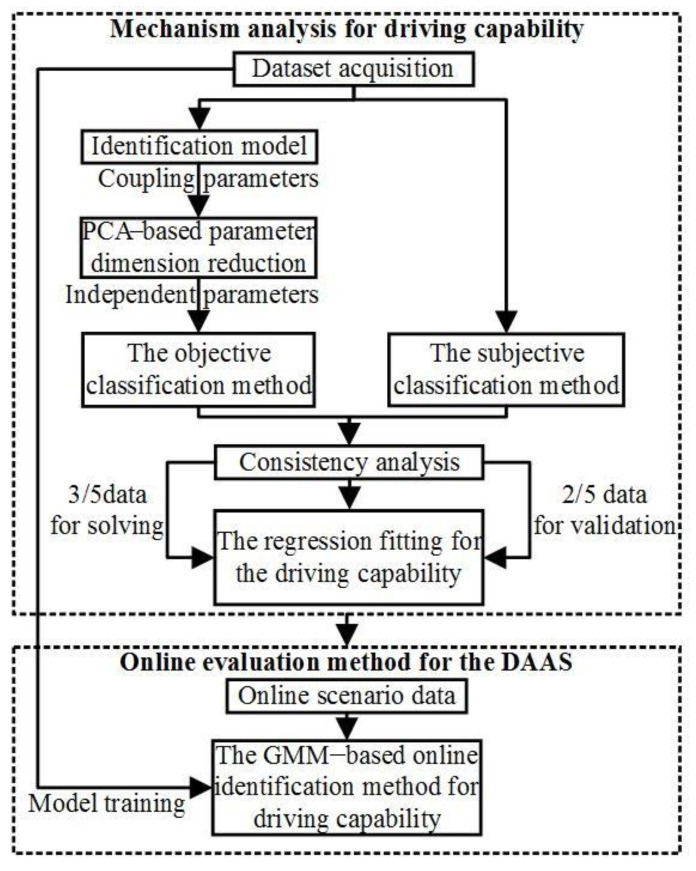
The analysis and evaluation framework of driver’s driving capability.

The mechanism analysis and evaluation framework of the driver’s driving capability is established and shown in Figure 3. The characteristics and mechanism of the driving capability are analyzed offline, and its evaluation method is conducted based on the online Gaussian mixture model (GMM) and introduced in detail in the DAAS in Section 3.

**Figure 4:**
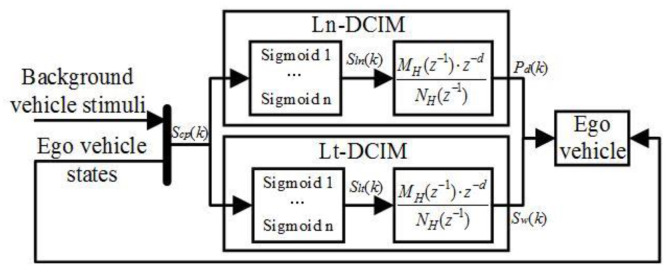
The Hammerstein identification process is based on the offline identification model of driving capability.

The Hammerstein identification process is proposed as the driving capability identification model (DCIM) in view of its conforming with the time-varying, high-order nonlinear, and dynamic features of the driving capability, as shown in Figure 4. As the set of longitudinal and lateral identification models (LnDCIM and LtDCIM), the DCIM consists of the static nonlinear and dynamic linear elements in series.

The static nonlinear and the dynamic linear elements can be expressed as follows:

(18) $$M_{H}(z^{-1})\cdot C_{p}(k)=N_{H}(z^{-1})\cdot z^{-d}\cdot S(k)$$

(19) $$\{\begin{array}{c} M_{H}(z^{-1})=1+m_{H,1}\cdot z^{-1}+\ldots +m_{H,q}\cdot z^{-i}\\ N_{H}(z^{-1})=n_{H,1}\cdot z^{-1}+\ldots +n_{H,n}\cdot z^{-j} \end{array}$$

where *C_p_*(*k*) consists of the pedal signal *P_d_(k)* and the steering angle signal *S_w_*(*k*)*, S*(*k*) is the set {*S_ln_*(*k*)*, S_lt_*(*k*)} in the static nonlinear element of DCIM, *q* and *n* are orders in the dynamic linear element, and d is the input delay order defined as the integer multiple of the sampling time.

As the key data to reveal the intrinsic attributes of driving capability, the parameters of the DCIM model need to be decoupled to avoid data oversaturation in the regression fitting. The principal component analysis (PCA) method [35] is adopted to decouple and reduce the key parameter dimension in the DCIM. The dataset *P_r_* with *ζ*-dimension parameters and U-dimension observation variables can be expressed as follows:

(20) $$P_{r}=\left[\begin{array}{cccc} p_{r,11} & p_{r,12} & \cdots & p_{r,1U}\\ p_{r,21} & p_{r,22} & \cdots & p_{r,2U}\\ \vdots & \vdots & \vdots & \vdots \\ p_{r,\varsigma 1} & p_{r,\varsigma 2} & \cdots & p_{r,\varsigma U} \end{array}\right]=[p_{r,1},p_{r,2},\ldots ,p_{r,U}]$$

The contribution rate of the principal component is defined as the percentage of the sum of the eigenvalues of the first *q* principal components and the sum of all ones, and the cumulative contribution rate *Q_q_* is as follows:

(21) $$Q_{\psi }=\sum_{i=1}^{\psi }\lambda_{i}/\sum_{i=1}^{U}\lambda_{i}$$

The *ψ* corresponding to *Q_ψ_* ≥ 85% is taken as the independent component number, and the principal component matrix *P_L_* with *ψ* × *U* dimensions can be obtained. The driving capability needs to be classified as the following model set based on the objective and subjective methods.

(22) $$D_{cap}=\{Excellent,Strong,Medium,Weak,Poor\}$$

The objective classification method consists of the particle clustering and the mapping process from the clustering results to *D_cap_*, and the subjective method is based on scale analysis. The element in the clustering set *O_P,ru_* that has the largest intersection with the specific one in the scale analysis set *S_P,ij_*, which is the driving capability element of the same type.

(23) $$CL_{P}=\{CL_{P,ry},r=1,\ldots ,5,\sum_{i}^{5}y=e\leq U\}$$

(24) $$CL_{P,ry}=\{O_{P,ru}\cap S_{P,ij},\max y,r=1,\ldots ,5\}$$

The driving capability evaluation equation (DCEE) is as follows:

(25) $$CL_{P}={\tilde{P}}_{L}\cdot \rho$$

where ${\tilde{P}}_{L}$ is the subset of *P_L_*, and *ρ* is the regression coefficient.

(26) $$\begin{array}{l} {\tilde{P}}_{L}={\left[\begin{array}{cccc} p_{l,11} & p_{l,12} & \cdots & p_{l,1u}\\ p_{l,21} & p_{l,22} & \cdots & p_{l,2u}\\ \vdots & \vdots & \vdots & \vdots \\ p_{l,q1} & p_{l,q2} & \cdots & p_{l,qu} \end{array}\right]}^{T};\rho =\left[\begin{array}{c} \rho_{1}\\ \rho_{2}\\ \vdots \\ \rho_{m} \end{array}\right];CL_{P}=\left[\begin{array}{c} CL_{P,1}\\ CL_{P,2}\\ \vdots \\ CL_{P,u} \end{array}\right];\\ CL_{P,i}=R\in \{1,2,3,4,5\},i=1,2,\ldots u;\;\;u\leq U \end{array}$$

### 2.3. The Characterization and Evaluation Method for Driving Styles

**Figure 5:**
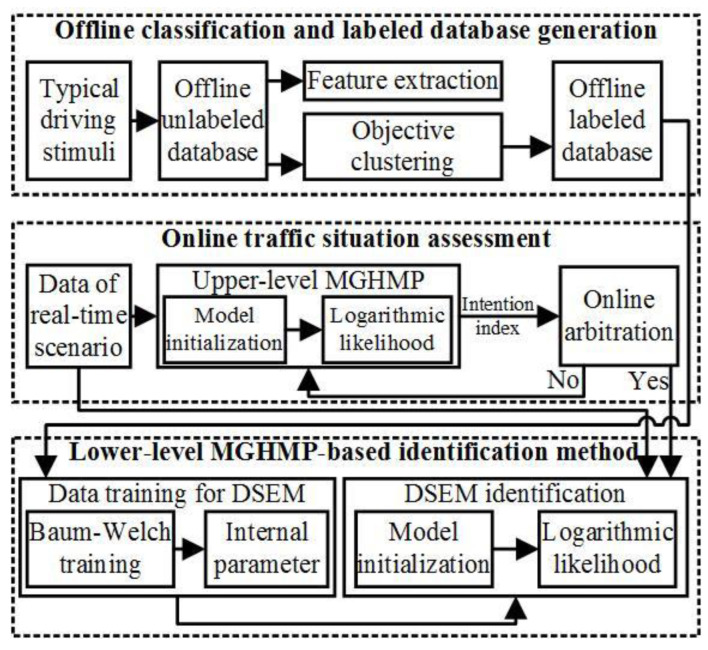
The characterization and evaluation framework of drivers’ driving styles.

The characterization and evaluation framework of drivers’ driving styles is shown in Figure 5. Accounting for classification application and computation complexities, the driving styles can be labeled as the offline database *D_Sty_* and are proposed to be classified into three types as follows:

(27) $$D_{Sty}=\left\{Steady\;type,general\;type,radical\;type\right\}$$

The driving style classification model (DSCM) combines the subjective and objective methods to approximate the truth value of the driving styles. Particle swarm clustering (PSC) is proposed as the objective classification method, and features of drivers’ driving styles are extracted as the physical property and model inputs, which can be expressed as follows:

(28) $$a_{\omega }={\left[\int_{f_{0}}^{f_{0}+F}PSD\left(f\right)df\right]}^{\frac{1}{2}}$$

where *a_ω_* is the root mean square of acceleration, *f*_0_ and *F* are the initial frequency and the integrating frequency range, and *PSD*(*f*) is the power spectral density of acceleration of the ego vehicle. The driver reaction time *T_s_* represents the driver’s sensitivity to the stimuli of the current situation and can be expressed as follows:

(29) $$T_{s}=T_{0},when\;\Delta v_{fir}=\frac{v_{ego}\left(T_{0}+n_{p}\cdot t_{sp}\right)-v_{ego}\left(T_{0}\right)}{n_{p}\cdot t_{sp}}\geq \Delta v_{0}$$

where *t_sp_* is the sampling period, *n_p_* is the sampling number, *v_ego_* is the velocity of the ego vehicle, Δ*v*_0_ is the velocity threshold, and *T*_0_ is the instant that *v_ego_* achieves Δ*v*_0_ for the first time. The time headway *T_f_* represents the approaching degree of the ego vehicle relative to the surrounding situation and is as follows:

(30) $$\left[\begin{array}{c} T_{f,\ln}\\ T_{f,lt} \end{array}\right]=\left[\begin{array}{c} D_{avg}/V_{avg}\\ D_{ins}/V_{ins} \end{array}\right]$$

where *D_avg_* and *D_ins_* are the average relative distance and the instant relative distance when the ego vehicle steers, and *V_avg_* and *V_ins_* are the corresponding relative velocities.

Each element in *D_Sty_* can be modeled as the DSCM, which can be designed as the function consisting of the mean and variance of the *a_ω_*, *T_s_,* and *T_f_* as follows:

(31) $$DSCM_{i}=\left(a_{\omega Mi},a_{\omega Vi},T_{sMi},T_{sVi},T_{fMi},T_{fVi}\right)\sim D_{sty},i=1,2,3$$

where variables with subscript *M* are those of their mean, and the ones with subscript *V* are those of their variance. The DSCM consists of the PSC and the mapping process between clustering centers and *D_Sty_*. The number of the clustering center is 3, and the mapping relation is as follows:

(32) $$\begin{array}{l} D_{Ag,i}=\omega_{1}\cdot \frac{Ca_{\omega Mi}}{CT_{sMi}\cdot CT_{fMi}}+\\ \omega_{2}\cdot \left(Ca_{\omega Vi}\cdot CT_{sVi}\cdot CT_{fVi}\right),i=1,2,3 \end{array}$$

where [*C_aωM_*, *C_TsM_*, *C_TfM_*, *C_aωV_*, *C_TsV_*, *C_TfV_*] is the clustering center of the DSCM, *ω*_1_ and *ω*_2_ are the weight coefficients, and *D_ag_* is the radicalization factor. The clustering center with the maximum of *D_ag_* is that of the radical type, and that with the minimum is that of the steady type.

The questionnaire method [36] is proposed as the subjective classification method and consists of five comfort degrees when respondents are passengers or drivers, respectively. The Cronbach α is used as the scale of confidence to verify the reliability and stability of the results as follows:

(33) $$\alpha =\frac{K}{K-1}\left(1-\frac{\sum_{i=1}^{K}\sigma_{i}^{2}}{\sigma_{Total}^{2}}\right)$$

where *K* is the question number, *σ_i_* and *σ_total_* are standard deviations of the score of the *ith* question and the total score of all the questions, respectively. The questionnaire for driving styles is shown in Table 1.

The traffic situation assessment will be introduced in detail in the intention-aware MOMDP framework in Section 3. Drivers’ operating signals, all states of the ego vehicle, and their relative states to the surrounding traffic are continuous time series and affect the states at the next moment. Therefore, the multi-dimension Gaussian hidden Markov process (MGHMP) with a set of hidden states *q_t_* and a corresponding set of *κ* possible observations is proposed as the driving style evaluation model (DSEM) as follows:

(34) $$\pi =\left\{\pi_{i}\right\}$$

(35) $$\pi_{i}=P\left(q_{1}=i\right),1\leq i\leq N$$

where *π* is the initial state distribution, and the state transition probability matrix A is as follows:

(36) $$a_{ij}=P\left(q_{t+1}=j|q_{t}=i\right),1\leq i,j\leq N$$

(37) $$a_{ij}=P\left(q_{t+1}=j|q_{t}=i\right),1\leq i,j\leq N$$

The set of observable sequences *O* = {*V_i_*, *i* = 1, 2,…, *κ*}, where *V* is the possible observation. The observation probability matrix of the *j*^th^ state is *B* = {*b_j_*(*O*)} and *b_j_*(*O*) is as follows:

(38) $$b_{j}\left(O\right)=\sum_{k=1}^{M}c_{jk}N\left(O|\mu_{jk},\Sigma_{jk}\right),1\leq j\leq N$$

(39) $$\int_{-\infty }^{+\infty }b_{j}\left(O\right)dO=1,1\leq j\leq N$$

where *c^jk^* is the *k*^th^ mixed-weight coefficient in the *j*^th^ state, and *M* is the Gaussian mixture number. N(*O*|*μ_jk_*, *Σ_jk_*) is the Gaussian probability density function with mean *μ* and covariance Σ. *c_jk_* is as follows:

(40) $$\sum_{k=1}^{M}c_{jk}=1,c_{jk}\geq 0,1\leq j\leq N,1\leq k\leq M$$

DSEM can be defined as a tuple *λ* with *N* states.

(41) $$\lambda =\left(\pi ,T_{r},c,\mu ,\Sigma \right)$$

Each DSEM is calculated as the logarithmic likelihood as follows, and the maximum likelihood in DSEM is mapped to the corresponding driving style.

(42) Loglik(θ)=ln[P(O|λ)]

## 3. Personalized Shared Control Strategy for the Automated Vehicle

Personalized shared control aims at improving driving safety and comfort and minimizing the driving load, which should be the optimal driving control match between the driver and the autonomous system. The personalized paradigm and its evaluation criteria are established for the proposed shared control system.

### 3.1. The Framework of the Personalized Shared Control

The strong coupling between the shared control and human–vehicle–scenario system is determined by the system function of the shared control. Therefore, the mechanism analysis of the holonomic system X with the human–controller–vehicle–scenario system is the development infrastructure of shared control, which can be expressed as follows:

(43) X={E,V,D,M,f(E,V,D,M)}

where *E* is the stimulating system, *D* is the driver system, *V* is the vehicle system, *M* is the shared control system, which consists of the personalized system *H_s_* and driving authority allocation system *D_as_*, and *f*(*E*, *V*, *D*, *M*) is the coupling function.

**Figure 6:**
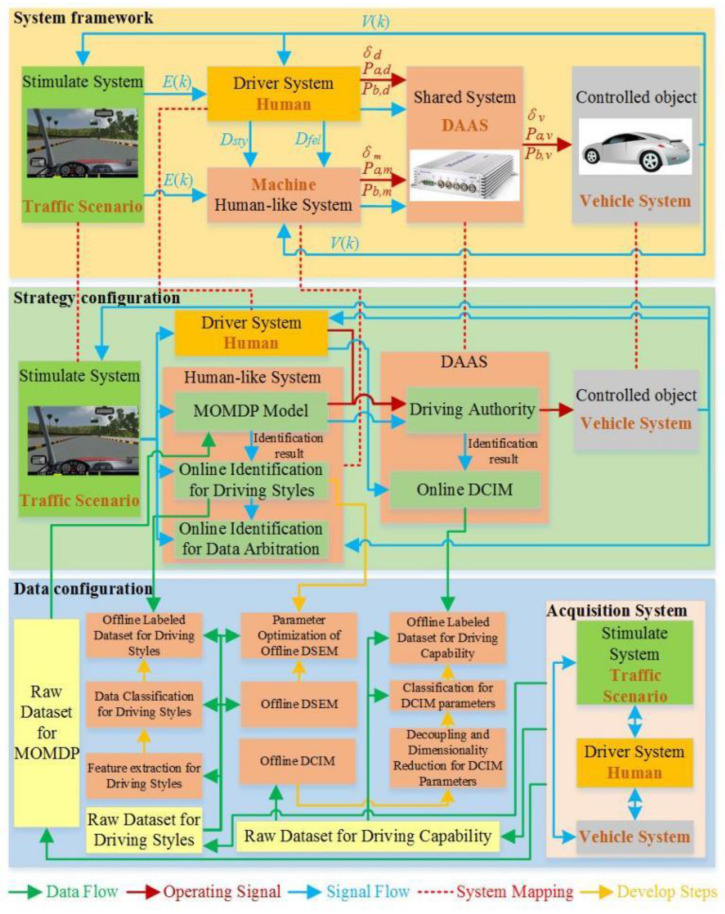
The framework of the personalized shared control.

*D* and *M* comprehend the current driving situation of *E* and adjust the operation mode applied to *V* based on their status feedback and form the cooperative mode by overcoming the inconsistent understanding of *E*. The framework of the personalized shared control consisting of the system, strategy, and data configurations is developed based on system features of X and large-scale data acquisition and training methods, as shown in Figure 6. The *H_s_* outputs personalized operation signals based on the *D_sty_* type, and operation and status from both *D* and *H_s_* are sent to *D,* and the driving authority is obtained based on the DAAS. The closed loop X is formed when states of *V* are changed and sent to *E*. The *M* outputs the online result of the *D_sty_* type, the real-time result of *D_cap_*, and the MOMDP-based decision-making result, which is based on the offline raw dataset, the offline database, and the online system states.

### 3.2. The DAAS and Evaluation Criteria for Shared Control

The *D_as_* arbitrates the driving authority between *D* and *H_s_* and outputs the desired control signals to *V*. The *D_as_* can be expressed as follows:

(44) $$D_{as}=f\left\{D_{f},E_{f}\left(O_{Ef},T_{Ef}\right),\Lambda \left(D_{f},E_{f}\right),\Lambda \left(D,H_{s}\right)\right\}$$

where *D_f_* is the human factor in *D*, *E_f_* is the situation factor in *E*, which is affected by the observability factor *O_Ef_* and the key situation factor *T_Ef_*, and Λ is the coupling effect function.

The *D_as_* in the indirect shared control has advantages in overcoming structure conflict of human–machine operation and improving human comfort, and is proposed as the DAAS in *M as* follows:

(45) $$C=\left\{\delta_{c},P_{a,c},P_{b,c}\right\}=\vartheta C_{d}+\left(1-\vartheta \right)C_{m}$$

where the control signal *C* consists of steer signal *δ_c_*, driving signal *P_a,c_*, and braking signal *P_b,c_*; *C_d_* and *C_m_* are operation signals of *D* and *H_s_*, respectively; and *ϑ* is the allocation coefficient, which determines the driving authority. The online evaluation result of driving capability is taken as *ϑ*, given its maneuverability to determine the control of *D*. The mapping relation from *D_cap_* = {1, 2, 3, 4, 5} to *ϑ* ∈ [0, 1] is *ϑ* = 0.25*D_cap_* − 0.25.

The DAAS is based on the GMM with an offline classification database of driving capability. The GMM consists of multiple single-Gaussian probability distribution models, and their probability density can be described by the Gaussian density function *g*(*x*, *μ*, *Σ*) as follows:

(46) $$g\left(x;\mu ,\Sigma \right)=\frac{1}{\sqrt{{\left(2\pi \right)}^{d}\left|\Sigma \right|}}\exp\left[-\frac{1}{2}{\left(x-\mu \right)}^{T}\Sigma^{-1}\left(x-\mu \right)\right]$$

where *x* is the random vector, *μ* is the mean vector, Σ is the covariance matrix, and *d* is the dimension of the random vector. Multidimensional Gaussian probability density functions *p*(*x*) need to be superimposed in the DSIM with multidimensional state inputs as follows:

(47) $$p\left(x\right)=\sum_{j=1}^{M}\alpha_{j}\cdot g_{j}\left(\theta_{j}\right),\;where\;\sum_{j=1}^{M}\alpha_{j}=1$$

where *x_j_*, *μ_j_*, and *Σ_j_* are the *j*^th^ random vector, mean vector, and covariance matrix, respectively, and *α_j_* is the weight coefficient. The likelihood function of N-dimensional sample *XG* is as follows:

(48) $$l\left(XG|\Theta \right)=\sum_{i=1}^{N}\log\sum_{j=1}^{M}\alpha_{j}\cdot g_{j}\left(\theta_{j}\right)$$

where *θ_j_* = (*x_j_*, *μ_j_*, Σ*_j_*), Θ = (*θ_1_*,…, *θ_M_*). The EM algorithm is proposed to train (*α*, *μ*, Σ) and consists of the E and *M* steps.

Accurate and objective evaluation criteria need to be established for shared control to evaluate the system’s performance. The system performance index of *M* is as follows:

(49) $$\Xi_{i=1,\ldots ,n}=\varepsilon_{1}\eta_{ds}+\varepsilon_{2}\eta_{cw}+\varepsilon_{3}\eta_{dc}\;where\;\varepsilon_{1}+\varepsilon_{2}+\varepsilon_{3}=1$$

where Ξ is the composite index of shared control, and the subscript *i* is the number of *E*. Ξ consists of the safety index *ŋ_ds_*, the driving load index *ŋ_cw_*, and comfort index *ŋ_dc_*, and *ε*_1_, *ε*_2_, and *ε*_3_ are the corresponding weight coefficients. In order to reveal the system’s performance, values of *ε*_1_ and *ε*_3_ should be higher than *ε_2_* and can be set as {*ε*_1_, *ε*_2_, *ε*_3_} = {0.4, 0.2, 0.4}. The *ŋ_ds_*, *ŋ_cw_*, and *ŋ_dc_* in the longitudinal and lateral scenarios are as follows:

(50) $$\left[\begin{array}{c} \eta_{ds}\\ \eta_{cw}\\ \eta_{dc} \end{array}\right]=\left[\begin{array}{c} {\left(\int_{0}^{T}D_{cap,ln}dt\right)}^{-1}\\ \int_{0}^{T}\left[\left(P_{ac}+{\dot{P}}_{ac}\right)+\left(P_{br}+{\dot{P}}_{br}\right)\right]dt\\ N\left(a_{\omega M,h}-a_{\omega M,j}\right)+N\left(T_{sM,h}-T_{sM,j}\right)+N\left(T_{fM,h}-T_{fM,j}\right) \end{array}\right]$$

(51) $$\left[\begin{array}{c} \eta_{ds}\\ \eta_{cw}\\ \eta_{dc} \end{array}\right]=\left[\begin{array}{c} {\left(\int_{0}^{T}D_{cap,lt}dt\right)}^{-1}\\ \int_{0}^{T}\left(S_{w}+{\dot{S}}_{w}\right)dt\\ N\left(t_{\max,h}-t_{\max,j}\right)+N\left(T_{sM,h}-T_{sM,j}\right)+N\left(T_{fM,h}-T_{fM,j}\right) \end{array}\right]$$

where *D_cap_*_,*ln*_ and *Dc_ap,lt_* are the longitudinal and lateral driving capabilities, *P_ac_* and *P_br_* are the positions of the gas and braking pedals, and *S_w_* is the angle of the steering wheel. The variables corresponding to the subscript *h* are the statistics of the driving features in the case that *ϑ* = 1 and N is the normalization operator, which represents the deviation degree between the statistics and the specified value of the driver. *Ɩ_max_* is the maximum curvature, and the subscript *j* = 1, 2, 3 represents three modes: the shared control, human, and automated driving modes.

### 3.3. The Personalized Decision-Making Logic in the Intention-Aware MOMDP Framework

**Figure 7:**
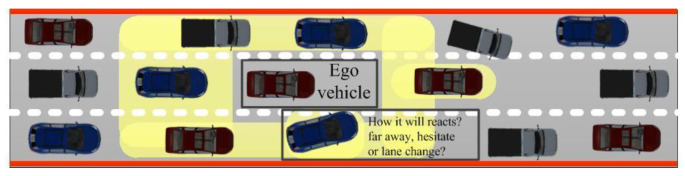
The coupling mechanism of motion intentions in micro-traffic scenarios.

The personalized decision-making logic is developed based on MOMDP to improve the acceptance and adaptation of shared control, and the intention-aware-based assessment method is proposed for the uncertainty in complex scenarios based on the MGHMP. Vehicles’ motion intention in the micro-traffic scenarios can be regarded as the set of longitudinal and lateral relative intentions in spatial evolution, and their reaction behaviors can be regarded as the system response to states *s*_0_ of the background vehicles and their relative states *ds*_0_ to the ego vehicle. The coupling mechanism of motion intentions in micro-traffic scenarios is shown in Figure 7. The driving motion intention model (DMIM) appears to be extended infinitely with an increase in the coupling region quantity. Therefore, the reactive motion intention model (RMIM) is proposed for adjacent background vehicles to improve model universality and controllability of the model scale.

The motion intention of background vehicles can be expressed as follows:

(52) $$I_{m}=\{I_{R},I_{D}\}=\{f_{R}(s_{0},ds_{0}),f_{D}(s_{0})\}$$

where *I_R_* is the motion intention set based on the RMIM, and *I_D_* is that based on the DMIM. The RMIM is the function of states *s*_0_ and *ds*_0,_ and the DMIM is the function of *s_0_* as follows:

(53) $$I_{R}=\{I_{FA},I_{HT},I_{NM},I_{CI}\},I_{D}=\{I_{LE},I_{RI},I_{FO}\}$$

where {*I_FA_*, *I_HT_*, *I_NM_*, *I_CI_*} are the intentions of bearing off, hesitation, maintaining, and approaching, and {*I_LE_*, *I_RI_*, *I_FO_*} are those of turning left, turning right, and keeping straight.

**Figure 8:**
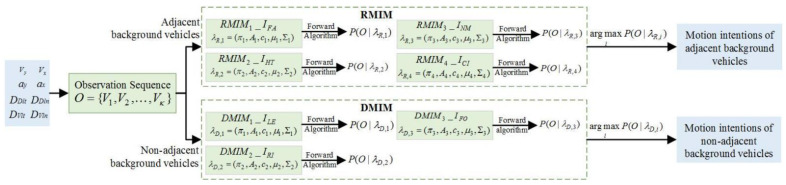
The framework of $I_{m}=\{I_{R},I_{D}\}=\{f_{R}(s_{0},ds_{0}),f_{D}(s_{0})\}$ for the online TSIM.

The observation sequences consisting of states *s*_0_ and *ds*_0_ are continuous time series and affect the states at the next moment. Therefore, the MGHMP with a set of hidden states *q_t_* and a corresponding set of *κ* possible observations is proposed as the online identification model of the traffic situation (TSIM) for both DMIM and RMIM, as shown in Figure 8. The derivation process of the TSIM is similar to that of the DSEM.

The MOMDP-based personalized decision-making process can be expressed as a tuple as follows:

{*S*, *A*, *T*, *Z*, *O*, *R*, *γ*} (54)

where *S* is the state space, *A* is the action space, *T*(*s′*, *s*, *a*) = Pr(*s′*|*s*, *a*): S × A × S is the transition function, *Z* is the observation space, and *O*(*z*, *s′*, *a*) = Pr(*z*|*s′*, *a*): *S* × *A* × *Z* is the observation function. *R*(*s*, *a*): *S* × *A* is the reward function, *γ* ∈ [0,1] is the discount factor. The uncertainty factor of the MOMDP is the motion intention of vehicles, and the belief *b* ∈ *B* and the Bayes rule-based updated belief *b′* = *τ*(*b*, *a*, *z*) is proposed for the uncertainty, where *B* is the belief set, and *τ* is the belief updating function, with *a* and observation *z* as follows:

(55) $$b^{\prime }\left(s^{\prime }\right)=\eta \cdot O\left(s^{\prime },a,z\right)\cdot \sum_{s\in S}T\left(s,a,s^{\prime }\right)b\left(s\right)$$

where *ŋ* is the normalization coefficient as follows:

(56) $$\eta ={\left(\sum_{s\in S}O\left(s^{\prime },a,z\right)\sum_{s\in S}T\left(s,a,s^{\prime }\right)b\left(s\right)\right)}^{-1}$$

The MOMDP-based personalized decision-making process aims at searching the strategy π* corresponding to maximize *R* as follows, where *π* is the mapping strategy with the specified action *a* = *π*(*b*) and *b*_0_ is the initial belief.

(57) $$\pi^{*}=\underset{\pi }{\arg\max}\left(E\left(\sum_{t=0}^{\infty }\gamma^{t}R\left(s_{t},\pi \left(b_{t}\right)\right)|\left(b_{0},\pi \right)\right)\right)$$

Abundant messages for the decision-making process should be contained in *S*, given the property of the Markov process, and can be expressed as follows:

(58) $$\{\begin{array}{l} S=\left\{X_{s},Y_{s}\right\}\;\;\;\;\;\;\;\;\;\;\;\;\;\;\;\;\;\;\;\;\;\;\;\;\\ X_{s}=\left[x,y,\theta ,V_{x},V_{y},a_{x},a_{y},Y_{aw}\right]\\ Y_{s}=I_{m}\;\;\;\;\;\;\;\;\;\;\;\;\;\;\;\;\;\;\;\;\;\;\;\;\;\;\;\;\;\;\; \end{array}$$

where [*x*, *y*, *θ*] is the vehicle position and [*V_x_*, *V_y_*, *a_x_*, *a_y_*, *Y_aw_*] are the velocity and acceleration in longitudinal and lateral directions and yaw velocity, respectively. The current state set *s* = [*s_ego_*, *s_t_*_1_, *s_t_*_2_,…, *s_tN_*], where *s_ego_* is the state of the ego vehicle, and the others are those of background vehicles. Therefore, the mixed observable MDP is established and consists of the complete observable state set *X_s_*, the inference-based state set *Y_s_*, and the action space *A* as follows:

(59) $$\begin{array}{l} A=\{a_{lon},a_{lat}\};\\ a_{lon}=[a_{lon,a},a_{lon,d},a_{lon,c}];\\ a_{lat}=[a_{lat,a},a_{lat,d},a_{lat,c}]; \end{array}$$

where [*a_ln_*, *a_lt_*] are the longitudinal and lateral actions, and the corresponding subscripts [*a*, *d*, *c*] are the acceleration, deceleration, and cruise control, respectively. The observation space *z* = [*z_ego_*, *z_t1_*, *z_t2_*,…, *z_tN_*], where *z_ego_* is that of the ego vehicle, and the others are those of background vehicles. The state transition model *T*(*s′*, *s*, *a*) = Pr(*s′*|*s*, *a*) predicts the dynamic randomness of the system affected by the ego vehicle and background vehicles given the current *s_ego_* and *a_ego_* as follows:

(60) $$\mathrm{Pr}(s^{\prime }|s,a)=\mathrm{Pr}(s_{ego}^{\prime }|s_{ego},a_{ego})\overset{N}{\underset{i=1}{\Pi }}\mathrm{Pr}(s_{i}^{\prime }|s_{i})$$

where *a_ego_* is the current action of the ego vehicle, Pr(*s′_ego_*|*s_ego_*, *a_ego_*) is the transition probability from the *s_ego_* and *a_ego_* to those of the next moment as per Equation (61), and $\mathrm{Pr}(s_{i}^{\prime }|s_{i})$ is the transition probability of background vehicles as per Equation (62).

(61) $$\left[\begin{array}{c} x^{\prime }\\ y^{\prime }\\ V_{x}^{\prime }\\ a_{x}^{\prime }\\ \theta^{\prime }\\ V_{y}^{\prime }\\ a_{y}^{\prime }\\ Y_{aw}^{\prime } \end{array}\right]=\left[\begin{array}{c} x\\ y\\ V_{x}\\ a_{x}\\ \theta \\ V_{y}\\ a_{y}\\ Y_{aw} \end{array}\right]+\left[\begin{array}{c} V_{x}\cdot \Delta t+\frac{1}{2}a_{lon}\cdot \Delta t^{2}\;\\ V_{y}\cdot \Delta t+\frac{1}{2}a_{lat}\cdot \Delta t^{2}\\ a_{lon}\cdot \Delta t\\ a_{lon}\\ 0\\ a_{lat}\cdot \Delta t\\ a_{lat}\\ 0 \end{array}\right]$$

(62) $$\mathrm{Pr}(s_{i}^{\prime }|s_{i})=\sum_{a_{i}}\mathrm{Pr}(s_{i}^{\prime }|s_{i},a_{i})\mathrm{Pr}(a_{i}|s_{i})$$

where $\mathrm{Pr}(s_{i}^{\prime }|s_{i},a_{i})$ and $\mathrm{Pr}(a_{i}|s_{i})$ need to be conducted as follows:

(63) $$\mathrm{Pr}(s_{i}^{\prime }|s_{i},a_{i})=\mathrm{Pr}(x_{i}^{\prime }|x_{i},a_{i})\mathrm{Pr}(I_{m,i}^{\prime }|x_{i}^{\prime },x_{i},I_{m,i},a_{i})$$

where $\mathrm{Pr}(x_{i}^{\prime }|x_{i},a_{i})$ can be expressed as Equation (59), and Pr(*s′_i_*|*s_i_*, *a_i_*) can be expressed as follows when *I_m_* is supposed to be maintained in the current moment and changes with the sample of the input data in the next moment.

(64) $$\mathrm{Pr}(a_{i}|s_{i})=\sum_{x_{ego}^{\prime }}\mathrm{Pr}(a_{i}|x_{ego}^{\prime },x_{i},I_{m,i})\mathrm{Pr}(x_{ego}^{\prime }|x_{i},I_{i})$$

where $\mathrm{Pr}(x_{ego}^{\prime }|x_{i},I_{m,i})$ can be obtained based on Equation (61) and Pr(*a_i_*|*x′_ego_*, *x_i_*, *I_m,i_*) can be conducted as follows, which is based on the deterministic model.

(65) $$\begin{array}{l} a_{i}=a_{i,low},\;\;if\;a_{i,comft}<a_{i,low}\\ a_{i}=\frac{a_{i,comft}+a_{i,low}}{2},\;\;\;\;if\;a_{i,comft}\geq a_{i,low} \end{array}$$

where *a_i,low_* and *a_i,comft_* are the lower and upper limits of acceleration. The observation model is the data sequence in the data acquisition and measurement process as follows:

(66) $$\begin{array}{l} \mathrm{Pr}(z|a,s^{\prime })]=\mathrm{Pr}(z_{ego}|s_{ego}^{\prime })\overset{N}{\underset{i=1}{\Pi }}\mathrm{Pr}(z_{i}|s_{i})\\ where\;\mathrm{Pr}(z_{ego}|s_{ego}^{\prime })\sim N(z_{ego}|x_{ego}^{\prime },\Sigma_{z_{ego}}) \end{array}$$

**Figure 9:**
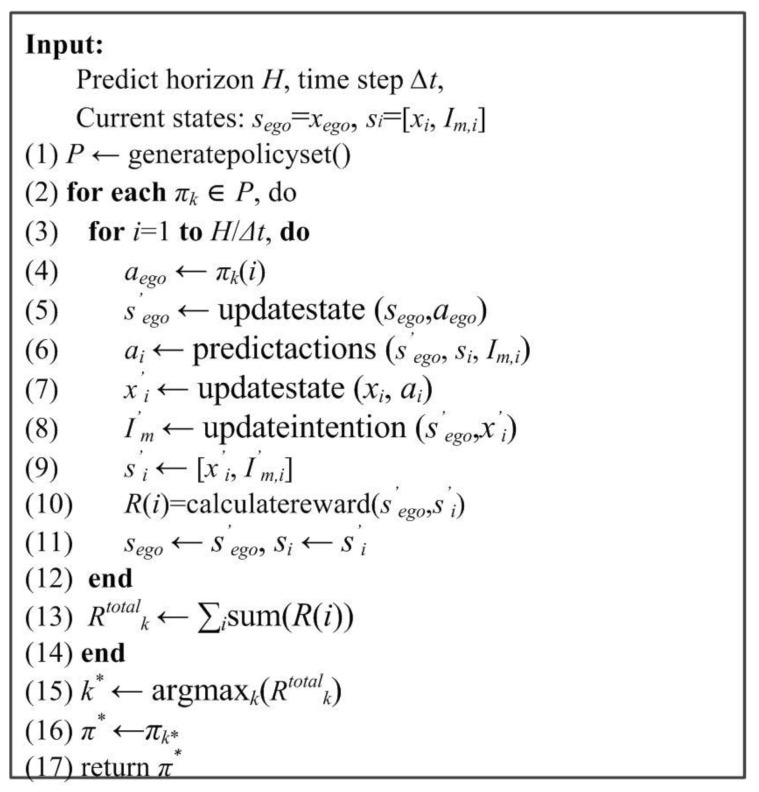
The policy selection pseudocode.

The reward function *R* is required to be mapped with personalized factors, obey the traffic rules, consider driving safety and comfort, and complete in the shortest time, as follows:

(67) $$\begin{array}{l} R\left(s,a\right)=\mu_{1}R_{saf}\left(s,a\right)+\mu_{2}R_{goal}\left(s,a\right)\\ +\mu_{3}R_{law}\left(s,a\right)+\mu_{4}R_{comf}\left(s,a\right) \end{array}$$

where [*R_saf_*, *R_goal_*, *R_law_*, *R_comf_*] are the safety, time, traffic rules, and comfort rewards, and [*μ*_1_, *μ*_2_, *μ*_3_, *μ*_4_] are the weight coefficients. The policy selection pseudocode is shown in Figure 9.

## 4. Experiment Platform and Scenario Generation

The evaluation process for the personalized shared control aims to verify the shared control performance, the decision-making logic, and the key human factors. The rationality and validity of personalized shared control for automated vehicles are proposed to be evaluated in the human-in-the-loop simulation platform and the field test platform based on the automated vehicle and the related testing scenarios.

### 4.1. The Real-Time Human-in-the-Loop Simulation Platform

**Figure 10:**
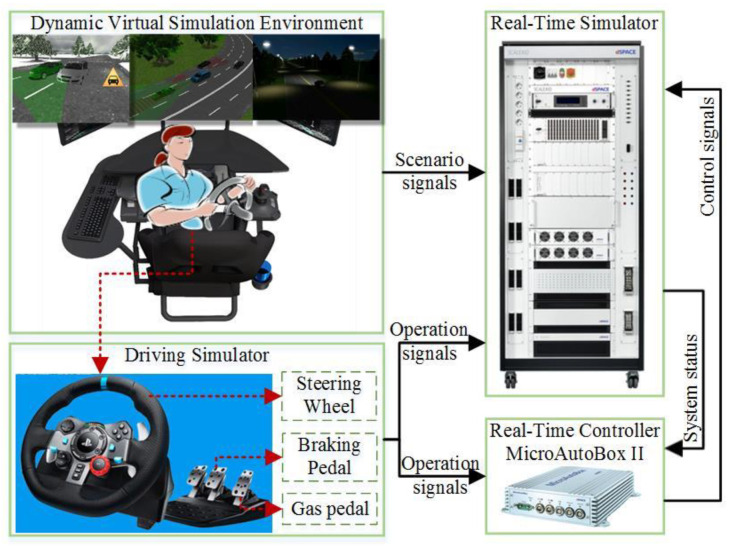
Human-in-the-loop real-time co-simulation platform.

The core of the personalized simulation platform is the driver and shared controller of the automated system, and the main components of the simulation platform are by-wire assembly and driving simulator. The human-in-the-loop co-simulation platform with a real-time concurrent pattern is shown in Figure 10, which consists of the virtual simulation environment, driving and real-time simulators, and the real-time controller. The shared control runs in dSPACE MicroAutoBox II from dSPACE GmbH in Paderborn, Germany, in real-time, and the driver operates the G29 driving simulator in a dynamic virtual simulation environment from Panosim 8.1. The vehicle dynamic model and traffic scenarios run in the dSPACE simulator, and the real-time controller receives the driver’s operation and system states and outputs the control signals as feedback.

### 4.2. The Automated Vehicle Platform for Shared Control

**Figure 11:**
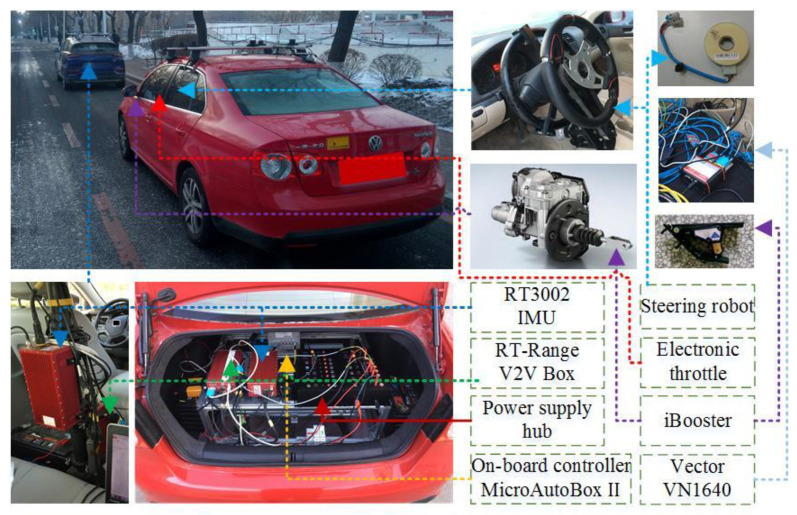
Field test platform for shared control.

The driver and on-board controller are key components in the field test. Therefore, the types of equipment in the field test platform for shared control consist of the on-board sensors, the on-board controller, the by-wire actuators, and the V2V communication equipment, as shown in Figure 11. The field test platform consists of an ego vehicle and a background vehicle; the RT3000 IMU measures vehicle states, and the RT-Range is equipped with V2V communication equipment. The on-board controller MicroAutoBox II achieves a real-time process for the shared control. The position sensor of the braking pedal and the angle sensor of the steering wheel are equipped to acquire the driver’s braking and steering signals, respectively. The steering robot, electronic throttle, and iBooster are used as the actuators for the shared control based on their by-wire performance, while the signals for the actuators are decoupled with those from the driver and the shared control.

### 4.3. Scenario Generation Results of Stimuli and RVRF for Human Factors

The natural driving scenario database with about 30,000 km and 66,600 scenario sections was established for the training and test sets for the RVRF. A total of 30 drivers, aged 25 to 40 years old with more than 10 driving years and a driving frequency of more than 14 h per week, were selected, as shown in Table 2. As shown in Table 2, typical driving scenarios were collected based on the road topology, and typical driving behavior was collected during the large-scale data acquisition process. Ωs under time slices of forthright and their *H_2D_mean_*(*E_m_*, *E_n_*) and *S_I_* were calculated. Scenario slices with *S_I_* > 95% were clustered as the same types, and the clustering results are shown in Table 2.

**Figure 12:**
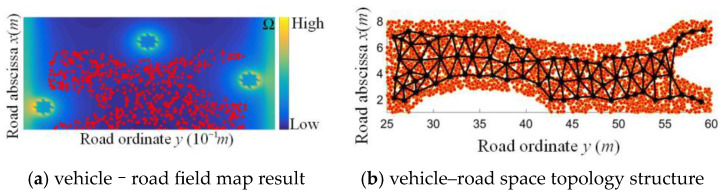
Topology structure results.

Three-lane, forthright scenarios without side parking are taken as an example for the verification of the RVRF. The ego vehicle enters into a low-field strength region of the vehicle–road space with particle nature and randomness, as shown in Figure 12a. Figure 12b is the extraction result of the topology structure in vehicle–road space, consisting of orange nodes for the scenario samples and a black topology structure. Errors’ RMSs of GNG with good convergence were calculated, as well.

**Figure 13:**
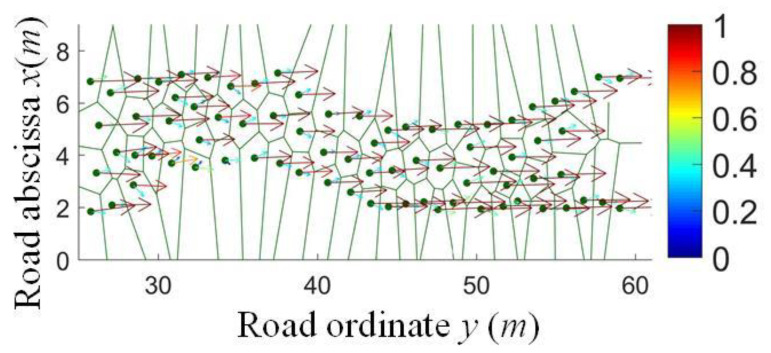
The result of the vehicle–road spatio-temporal states in the virtual micro RVRF.

**Figure 14:**
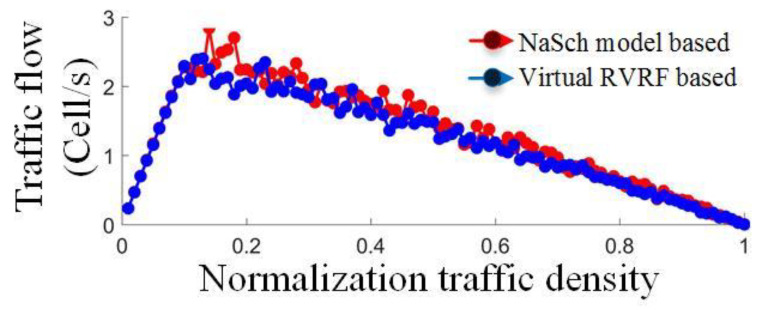
Comparison of traffic flow fluctuation results in the virtual macro RVRF.

A total of 3/5 data were taken as the training set, and the other 2/5 as the test set to achieve the accuracy verification results, and the vehicle–road spatio-temporal states based on Voronoi are shown in Figure 12, respectively. The node set and boundary set were obtained, and the position and states of the ego vehicle were determined by the RVRF, as shown in Figure 13; the probability of state transition between mutually exclusive regions is represented by an arrow vector colored with probability values. The imitative effect and volatility of the RVRF are compared with the NaSch model with a cell length of 1.5 m, sample period of 1 s, and vehicle length of 4.5 m. The macroscopic traffic flow results show high consistency between RVRF and NaSch models, as shown in Figure 14. According to the specific combination of *χ* and *ε*, the RVRF can be applied to different geographical or cultural contexts.

## 5. Experiment Verification and Performance Analysis

### 5.1. Analysis and Evaluation Results of Driving Capability

**Figure 15:**
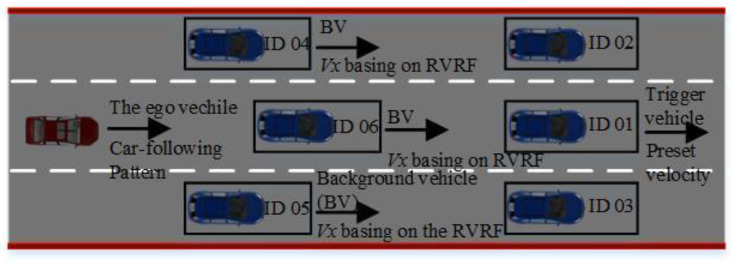
Configuration of longitudinal stimuli.

**Figure 16:**
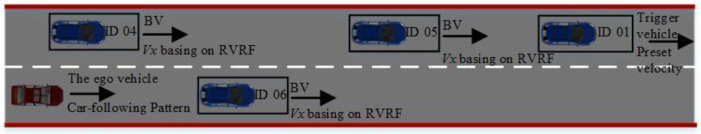
Configuration of lateral stimuli.

The stimuli with typical topology structures generated from the RVRF based on the scenario occurrence frequency from the naturalistic driving scenario database are proposed to reveal the mechanism of driving capability and to analyze its evaluation effectiveness, as shown in Figure 15 and Figure 16. Five drivers aged 25 to 40 years old with more than 10 driving years and a driving frequency of more than 14 h per week were chosen to conduct the continuous cyclic tests consisting of more than 6 h and 10 groups. A single test in the cyclic tests lasted from 10 s to 60 s; the end of the cyclic test ended when four continuous accidents occurred by the driver or the driver was unable to continue driving. The S-type function was taken as the static nonlinear element and orders *q* = 3, *n* = 3, *d* = 1 in the dynamic linear element.

**Figure 17:**
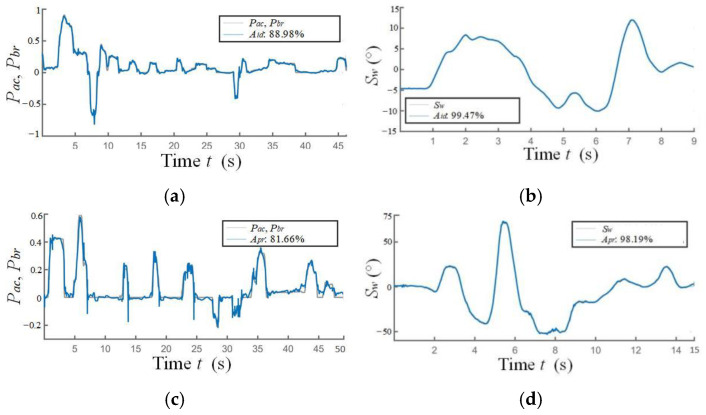
The identification and prediction results of typical longitudinal and lateral driving capabilities. (**a**) Aid of the longitudinal driving capability. (**b**) Aid of the lateral driving capability. (**c**) Apr in single tests NO. 166. (**d**) Apr in single tests NO. 223.

The results of the DCIM of the NO.1 driver are taken as an example, as shown in Figure 17. Figure 17a is the identification result Aid of the longitudinal driving capability in single test NO.165 of the NO.1 cyclic test. Figure 17b is that of the lateral driving capability in single test NO. 219 of the NO.1 cyclic test. Figure 17c,d are the prediction results for Apr of the single tests NO.166 and NO.223. The average accuracy of both Aid and Apr are above 85%, as shown in Table 3.

The longitudinal *ln* and lateral *lt* parameter configurations of the static nonlinear and dynamic linear elements are as follows:

(68) $$H_{\gamma }=\left\{SF_{\gamma },DY_{\gamma }\right\},\;\gamma =\left\{ln,lt\right\}$$

where *SF_γ_* and *DY_γ_* are the parameter dimensions in the S-type function and dynamic linear element, and *H_γ_* is the total dimension. *ψ* is calculated from the DCIM and is shown in Table 4.

**Figure 18:**
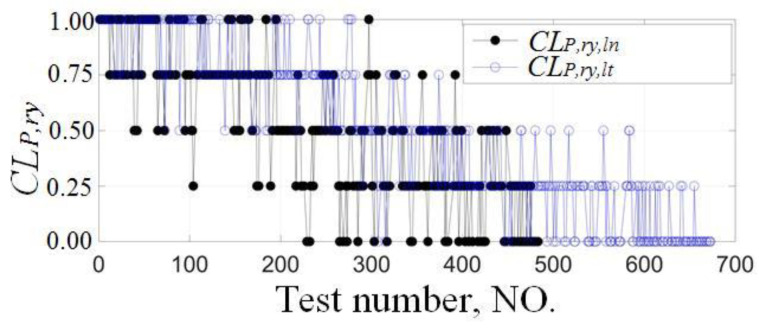
Classification results of the longitudinal and lateral driving capabilities.

The principal component dimensions of the longitudinal and lateral driving capabilities of the five tested drivers are {14(*ln*)/21(*lt*), 16/24, 14/22, 16/23, 15/22}, which are 1/3 to 1/4 of *H_γ_*. The clear and reasonable classification results of the driving capabilities are obtained by combining the subjective and objective classification methods, as shown in Figure 18.

**Figure 19:**
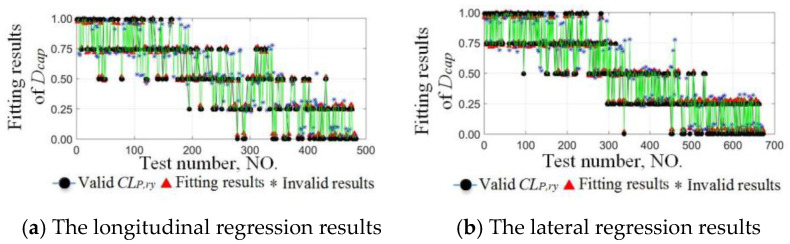
Regression results of typical longitudinal and lateral driving capabilities. Longitudinal and lateral prediction results of the 8th cyclic test of the NO.1 driver are shown in (**a**,**b**).

**Figure 20:**
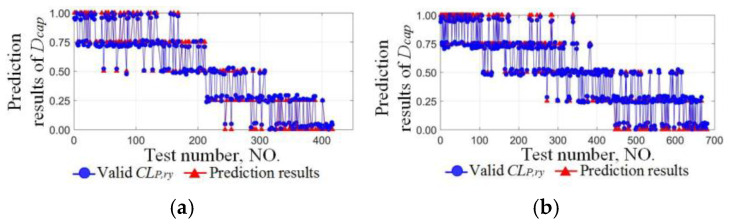
Fitting results of the driving capability evaluation equation. (**a**) The longitudinal effective regression results. (**b**) The lateral effective regression results.

Valid classification sets from six sets of the cyclic test were used as the training set, and those of the other four sets were used to verify the DCEE. As an example, the longitudinal and lateral training sets of the NO.1 driver are 1792 and 2710, and the verification set are 1105 and 1623, respectively. The longitudinal and lateral regression results are shown in Figure 19a,b, and the effective regression results are obtained based on the DCEE in Figure 20a,b.

It can be seen that both longitudinal and lateral driving capabilities represent their nonlinear, time-varying, and gradual features, as well as the volatility and randomness in several adjacent repeatability tests. These reasonable and effective results are obtained based on evaluation methods in conjunction with the DCIM and DCEE.

### 5.2. Analysis and Evaluation Results for Driving Styles

**Figure 21:**
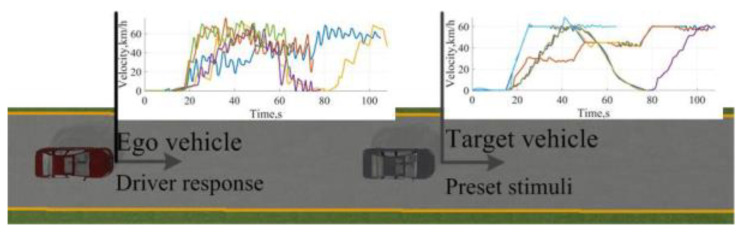
The configuration of the longitudinal stimuli.

**Figure 22:**
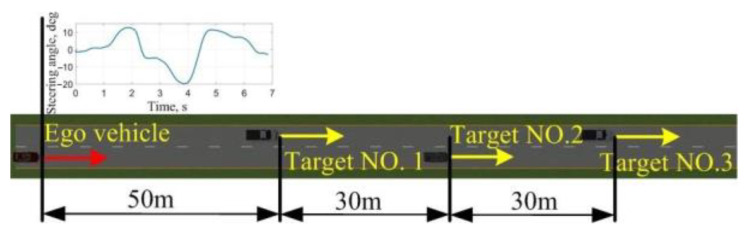
The configuration of the lateral stimuli.

The single source-based stimuli with an ego vehicle and a background vehicle (BV) are proposed in the analysis and evaluation for driving styles, as shown in Figure 21 and Figure 22. Based on the periodicity and mutability of the longitudinal velocity of the BV, the BV drives with preset velocity sequences, and the driver in the ego vehicle is required to keep a certain level of tension. The scenario of the urban structured road without side parking and 64 drivers consisting of 41 males and 23 females was chosen for data collection.

**Figure 23:**
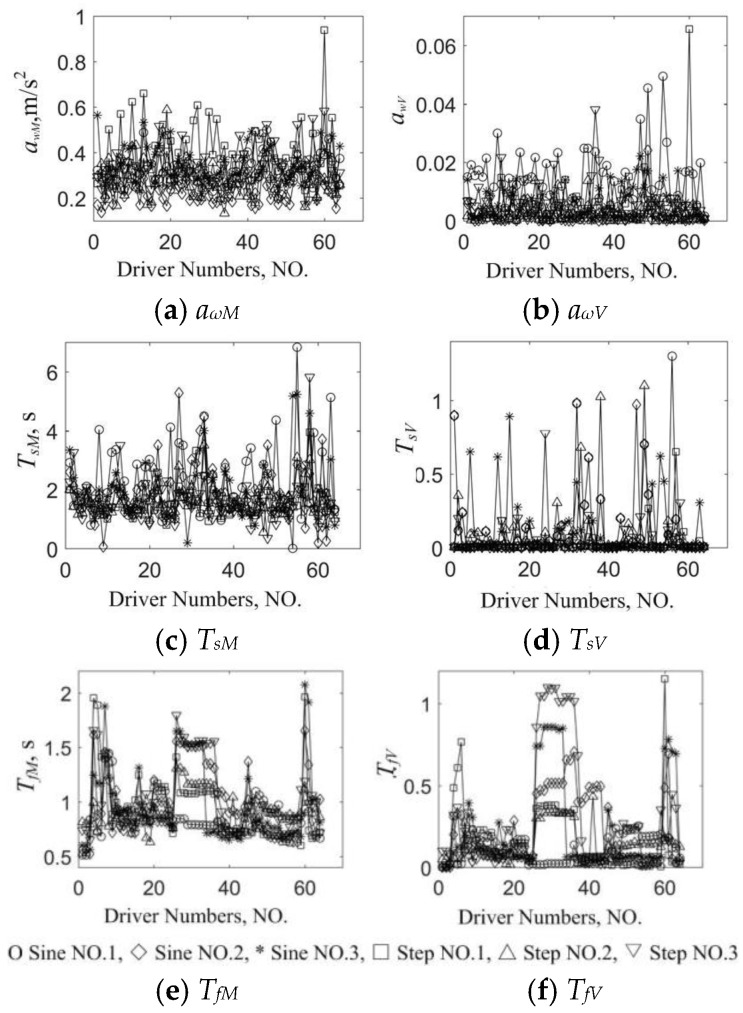
Extraction results of driving style features in typical longitudinal stimuli.

**Figure 24:**
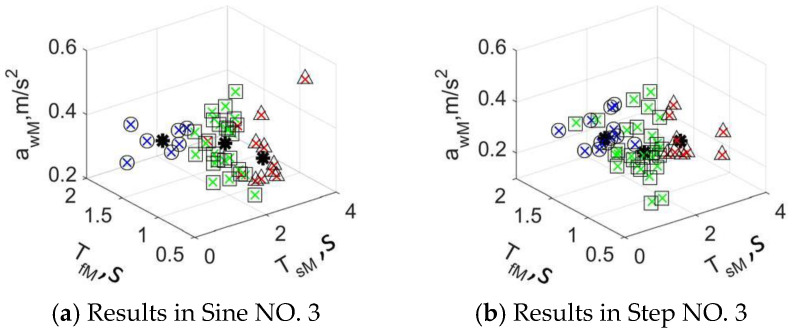
Classification results of driving styles in typical longitudinal stimuli.

Five data from each driver in a typical stimuli situation were selected, and 320 groups of data were taken as the classification sample. The objective features of the driving styles in typical stimuli are shown in Figure 23. Significant differences exist in *a_ωM_*, *T_sM_*, and *T_fM_*; ranges of *a_ωM_* and *T_sV_* are smaller, while *T_fM_* is bigger. The weight coefficients *ω_1_* = 10 and *ω_2_* = 10^3^, and the classification results of the driving styles are shown in Figure 24.

The questionnaire results of the subjective classification method are shown in Table 5. It can be seen that the more radical the driver is, the more steady he is to perceive others, and his passenger tends to perceive him as the radical one. The Sine 3 and Step 3 are proposed as the stimuli for the DSEM with *A_p_* = 30, *ω* = 2π/40, and *φ* = 0, *Γ* = 45, as well as the velocity sequence 0 → 20 → 50 → 70 → 50 → 20 → 0 km/h. A total of 192 groups of data were selected for model training, the Baum–Welch method [37] was proposed for the model training of the DSEM, and the verification data were the 128 groups. The input sets for the DSEM are shown in Table 6.

The four principal elements of the DSEM, the *M*, *N*, training period *TP*, and identification period *IP*, needed to be optimized, and the orthogonal experimental method is proposed with table L_9_(3^4^), where *M* = {4, 8, 12}, *N* = {4, 5, 6}, *TP* = {80, 90, 100}, and *IP* = {80, 85, 90}. Combining input sets and principal elements of the DSEM, optimal identification results are obtained, as shown in Table 7. It can be seen that the optimal input sets are those of 1 + 3 and 1 + 2 + 3 with the optimal principal element set {*M* = 12, *N* = 6, *TP* = 80, *IP* = 80}, and the identification accuracy is above 95%.

Based on the offline evaluation results, the online evaluation of driving styles was conducted with 50 drivers, and the naturalistic driving scenarios consisted of T-road, roundabouts and forthright with one to four lanes. The average online identification periods of driving styles are 1345 s of steady style, 1034 s of general style, and 762 s of radical style, which achieved an acceptable identification period with high accuracy.

### 5.3. Results of Personalized Shared Control for Automated Vehicles

**Figure 25:**
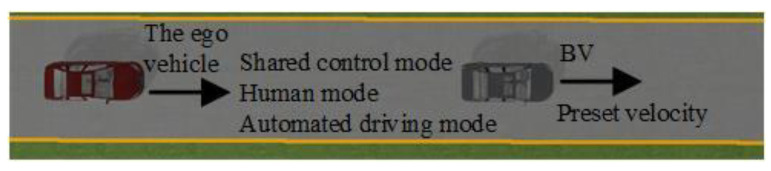
Configuration of the car-following forthright scenario.

**Figure 26:**
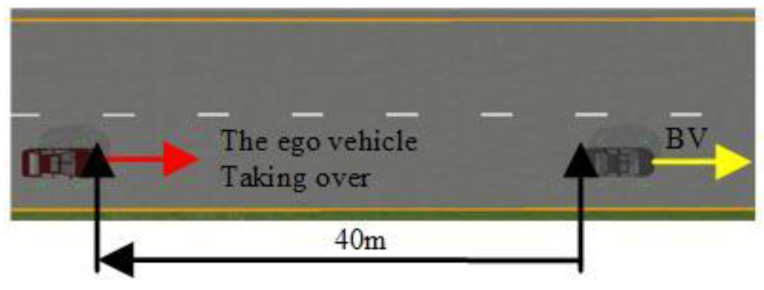
The configuration of the taking-over scenario in double lanes.

Personalized shared control was verified in the simulation platform and field test, respectively. The scenario configurations consisted of the car-following scenario in the forthright and taking-over scenario in the double lanes and comprised of 18 drivers, consisting of 9 males and 9 females aged from 24 to 45 years old with more than 14 driving hours per week, as shown in Figure 25 and Figure 26.

**Figure 27:**
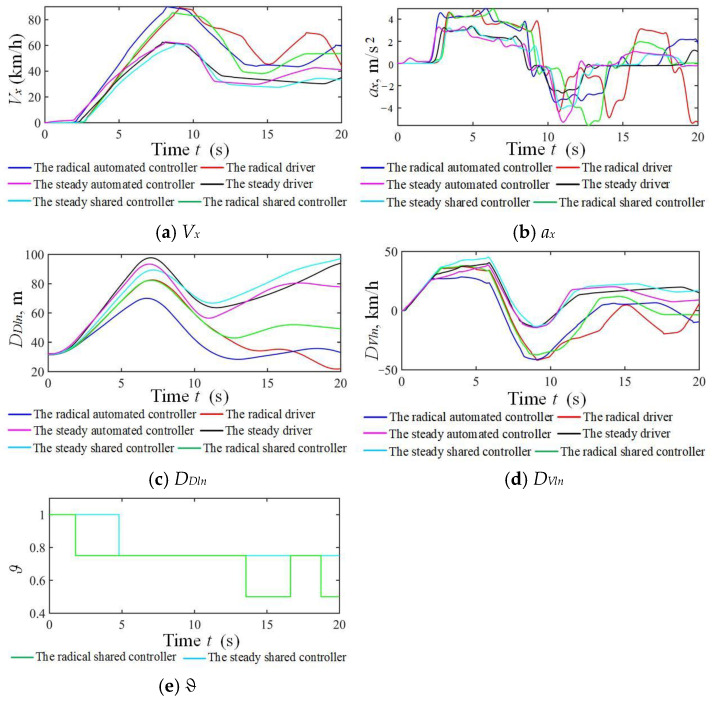
Simulation results of three modes corresponding to strong driving capability in the forthright.

**Figure 28:**
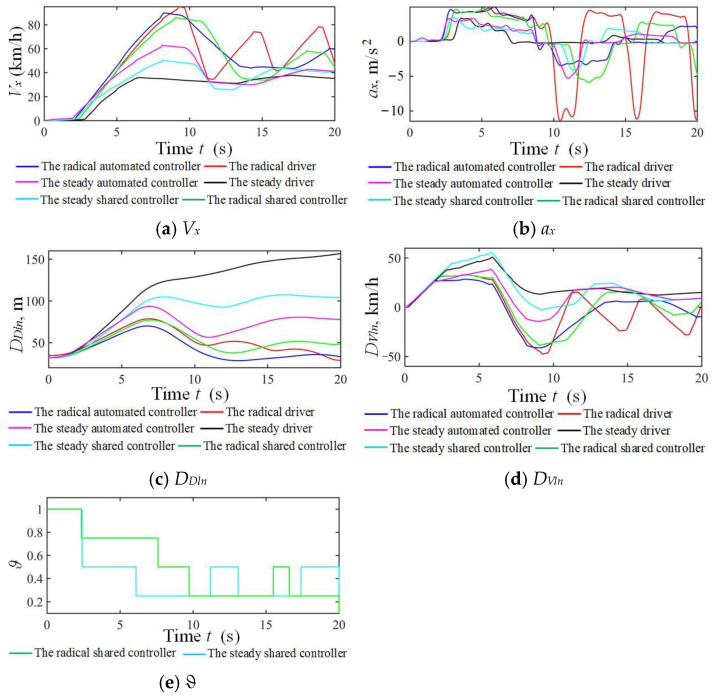
Simulation results of three modes corresponding to weak driving capability in the forthright.

The radical male driver NO.1 and steady male driver NO.7 are taken as examples in the car-following simulation scenario under strong and weak driving capabilities, as shown in Figure 27 and Figure 28. The car-following effects of the human mode and automated driving mode have significant differences under strong driving capability, and similarities exist in the car-following sequences in the three modes. The drivers obtained high and stable authority from the DAAS. In contrast, the radical driver obtained frequent velocity fluctuations and even conducted emergency braking, while the steady driver showed a tendency to drive away from a BV under weak driving capabilities. In this case, the drivers obtained lower authorities but the personalized system shared more from the DAAS to guarantee driving safety and improve the driver’s comfort.

The composite index Ξ of the NO.1 driver and the subindex are shown in Table 8. The shared control mode combines system features from both the human mode and automated driving mode, and the smallest and optimal Ξ can be obtained in different degrees of driving capabilities.

**Figure 29:**
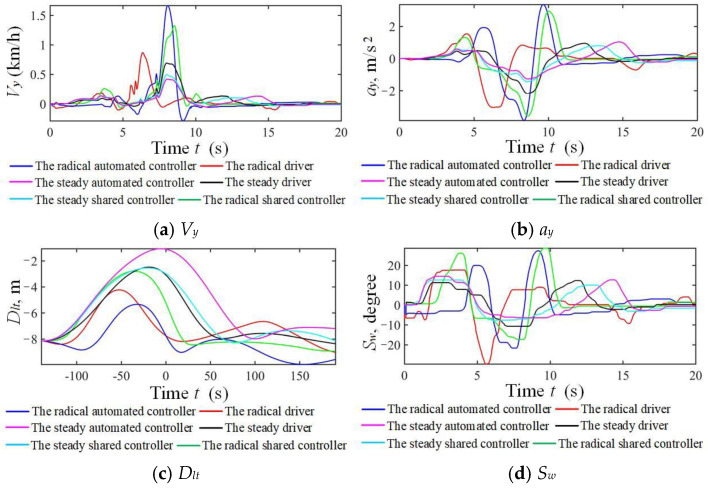

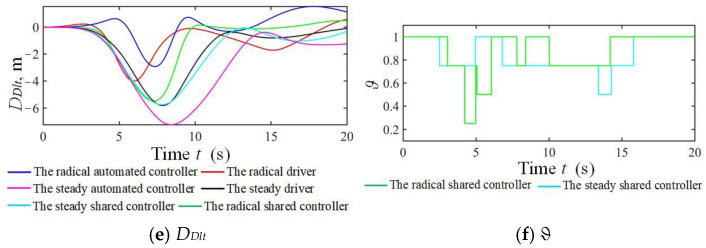
Simulation results of three modes corresponding to strong driving capability in double lanes.

**Figure 30:**
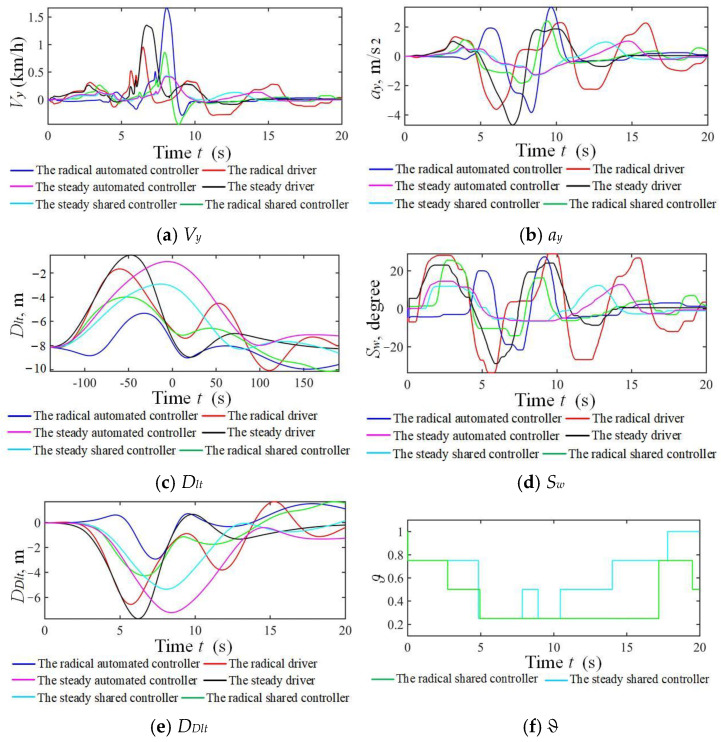
Simulation results of three modes corresponding to weak driving capability in double lanes.

The results for the NO.1 and NO.7 drivers in the taking-over simulation scenario under strong and weak driving capabilities are shown in Figure 29 and Figure 30. Similar results to those in the car-following scenario were obtained, except for a frequent steering wheel angle fluctuation from the radical driver. Shared control can guarantee driving safety, improve driver’s comfort, and reduce the workload in both longitudinal and lateral scenarios.

The Ξ of the NO.1 driver and the subindex are shown in Table 9. The smallest and optimal Ξs were obtained in different degrees of driving capabilities, as well.

Tests for the NO.1 and NO.7 drivers in the car-following field test under strong and weak driving capabilities were conducted. The preset velocity sequence of a BV is a step stimulate of 0 → 40 km/h, and similar results with those in the car-following simulation scenarios were obtained. The Ξ of the NO.1 driver and the subindex are shown in Table 10. The smallest and optimal Ξs were obtained, which is similar to those in the simulation results.

The radical male driver NO.1 and steady male driver NO.7 in the taking-over field test under strong and weak driving capabilities were conducted. The BV drove at a constant speed of 30 km/h, and the initial speed of the ego vehicle was 40 km/h. Similar results to those in the simulation scenario were obtained. Shared control can guarantee driving safety, improve driver’s comfort, and reduce workloads in both longitudinal and lateral scenarios. The Ξ of the NO.1 driver and the subindex are shown in Table 11, which shows the optimal performance of shared control. Furthermore, it is worth noting that although humans make moral decisions in principle, there are individual and cultural differences in moral justice [38], which should be the principle of observance during personalized shared control.

## 6. Conclusions

A personalized shared control, while considering drivers’ driving capabilities and styles, is proposed to improve the acceptance and adaptation of shared control by human drivers. As per theoretical and validation analyses, the following conclusions can be obtained:

The simulation scenario generation method for human factors was established. The RVRF theory reveals a wave-particle duality of macro- and micro-traffic flows. Stimuli and the RVRF achieved the ability to motivate driving capability and styles to the maximum extent. Drivers’ driving capability was defined and evaluated, the average accuracy of both Aid and Apr were above 85%, and its physical properties with nonlinearity, time gradient, randomness, and predictive ability were extracted and analyzed. Drivers’ driving styles were analyzed, characterized, and evaluated, and their accuracy was higher than 95% within a short identification period.

The MOMDP-based decision-making process shows advantages in dealing with uncertain motion intention and personalized logic. The personalized shared control system obtained excellent performance in both the human-in-the-loop simulation platform and field tests. The proposed system combines the randomness of human factor attributes in a single test, multi-objective optimization, and personalized characteristics of the driver and the automated driving system. Personalized shared control can achieve better performance in driving safety, comfort, and workload, corresponding to different driving capability degrees and driving style types than those only driven by human drivers or automated systems.

With the advantage of deep mixing decision-making between human and automated systems, personalized shared control will achieve better driver acceptance in future automated driving tasks.

## Figures and Tables

**Table 1:** Questionnaire for driving styles.

Questionnaire Content	1 Point	2 Point	3 Point	4 Point	5 Point
Feeling for the driver when as the passenger	Very soft	Soft	Comfort	Relatively radical	Radical
Feeling for passenger when as the driver	Very soft	Soft	Comfort	Relatively radical	Radical

**Table 2:** Clustering results of vehicle–road space situation.

Type	Main Case	Subcase	Results
Forthright	Lane number	(1) Side parking (2) Sparseness degree of the Surrounding traffic	182
Curve road	Curve and lanes		519
T-road	Angle and lanes		198
Intersection	Traffic lights		341
Roundabout	Curvature and driving actions		447

**Table 3:** Identification and prediction accuracies of DCIM.

Driver NO.	Average Aid	Average Apr
1	93.26%/95.92%	91.31%/95.16%
2	91.47%/96.77%	88.44%/95.93%
3	89.23%/94.81%	85.93%/93.44%
4	90.37%/96.42%	87.62%/96.21%
5	89.22%/93.01%	86.94%/92.89%
Total	90.710%/95.386%	88.048%/94.726%

**Table 4:** Parameter dimensions of DCIM.

Dimensions	*γ* = *ln*	*γ* = *lt*
*SF_γ_*	29	48
*DY_γ_*	20	30
Total	49	78

**Table 5:** Results of questionnaire classification method.

Driving Styles	Mean (Q1/Q2)	Variance (Q1/Q2)
Radical type	2.04/4.35	0.23/0.18
General type	3.32/3.53	0.39/0.19
Steady type	4.12/1.58	0.07/0.12
Total	3.44/3.34	0.29/0.17
The total variance is 0.79, and Cronbach α = 0.835 > 0.6		

**Table 6:** Input sets for the DSEM.

NO.	Input Set	States
1	Driver operation set	*P_ac_*, *P_ma_*
2	State set of the ego vehicle	*V_x_*, *a_x_*
3	Relative states	*D_Vln_*, *D_Aln_*

**Table 7:** Identification and accuracies in types stimuli.

Stimuli	Input Set to DSEM(%)					
	1	2	1 + 2	1 + 3	2 + 3	1 + 2 + 3
Sine3	92.2	85.9	93.8	99.2	96.9	98.4
Step3	93.0	91.4	92.2	100	96.1	99.2

**Table 8:** Results of Ξ and normalization subindex.

Modes	Car-Following in Single Lane			
	*ŋ_ds_*	*ŋ_cw_*	*ŋ_dc_*	Ξ
Human	0.54	0.83	0.31	0.506
Automated driving	0.16	0.52	0.36	0.312
Shared control	0.18	0.49	0.32	0.298

**Table 9:** Results of Ξ and normalization subindex.

Modes	Taking over in Double Lanes			
	*ŋ_ds_*	*ŋ_cw_*	*ŋ_dc_*	Ξ
Human	0.53	0.85	0.32	0.510
Automated driving	0.18	0.46	0.43	0.336
Shared control	0.20	0.47	0.35	0.314

**Table 10:** Results of Ξ and normalization subindex.

Modes	Car-Following in Single Lane			
	*ŋ_ds_*	*ŋ_cw_*	*ŋ_dc_*	Ξ
Human	0.63	0.77	0.28	0.518
Automated driving	0.15	0.39	0.36	0.282
Shared control	0.17	0.36	0.29	0.256

**Table 11:** Results of Ξ and normalization subindex.

Modes	Taking over in Double Lanes			
	*ŋ_ds_*	*ŋ_cw_*	*ŋ_dc_*	Ξ
Human	0.66	0.75	0.24	0.510
Automated driving	0.21	0.31	0.32	0.274
Shared control	0.24	0.29	0.25	0.254

## Data Availability

The authors do not have permission to share data.
